# A Novel Diosgenin-Based Liposome Delivery System Combined with Doxorubicin for Liver Cancer Therapy

**DOI:** 10.3390/pharmaceutics14081685

**Published:** 2022-08-12

**Authors:** Lixia Chen, Jinshuai Lan, Zhe Li, Ruifeng Zeng, Yu Wang, Lu Zhen, Haojieyin Jin, Yue Ding, Tong Zhang

**Affiliations:** 1 School of Pharmacy, Shanghai University of Traditional Chinese Medicine, Shanghai 201203, China; 2 Experiment Center of Teaching & Learning, Shanghai University of Traditional Chinese Medicine, Shanghai 201203, China

**Keywords:** diosgenin, doxorubicin, liposome, synergistic treatment, liver cancer

## Abstract

As a malignant tumor, liver cancer is mainly treated with chemotherapy, while chemotherapeutic drugs, such as doxorubicin (DOX), may lead to toxicity, drug resistance and poor prognosis. The targeted delivery systems of combining natural products and chemotherapeutic drugs are useful to eliminate cancers with reduced toxicity and increased efficiency. In this study, a diosgenin-based liposome loaded with DOX (Dios-DOX-LP) was developed for synergistic treatment of liver cancer, in which Dios not only replaced cholesterol as the membrane regulator to keep stability of liposomes, but also became the chemotherapy adjuvant of DOX for synergistic treatment. Dios-DOX-LP was characterized by particle size (99.4 ± 6.2 nm), zeta potential (−33.3 ± 2.5 mV), and entrapment efficiency (DOX: 98.77 ± 2.04%, Dios: 87.75 ± 2.93%), which had a good stability and slow-release effect. Compared with commercial DOX liposome (CHOL-DOX-LP), Dios-DOX-LP had an improved anti-tumor effect in vitro and in vivo by inducing the apoptosis and inhibiting the proliferation of the tumor cell, which was 1.6 times better than CHOL-DOX-LP in cytotoxicity, and had 78% of the tumor inhibition rate on tumor-bearing nude mice. Dios-DOX-LP provided a novel idea to achieve synergistic tumor treatment using diosgenin as a liposome material.

## 1. Introduction

Liver cancer still remains a global health challenge, and is one of the top ten cancer types in the world, which might have an estimated incidence of more than one million cases by 2025 [1,2]. The clinical treatments mainly include surgery, chemotherapy, immunotherapy, and so on. However, toxicity, serious side effects and drug resistance have led to the failure of most clinical chemotherapy [3,4]. Doxorubicin (DOX), a first-line chemotherapeutic drug, is commonly used in the treatment of tumors, especially in liver cancer [5]. Because of its cardiotoxicity and other side effects, liposomes containing DOX have been developed (Doxil^®^ and Caelyx^®^) for clinical tumor treatment. However, there are still various problems with single chemotherapy, such as drug resistance and low efficiency [6,7].

The combination of drugs, especially the synergy between natural products and chemotherapeutic drugs, has been a focus of research in recent years [3]. Natural products not only have the active anticancer properties, but also have relative safety in terms of drug discovery and development [8]. It was proved that natural compounds used with conventional chemotherapeutics at the same time might have better cytotoxicity [9], reduce chemoresistance [10], intensify the synergistic effects [11], or exert specific cytotoxic effects [12] on tumor cells. Diosgenin (Dios), a steroidal saponin extracted from the seeds of fenugreek (*Trigonella foenum graecum Linn*) and the roots of wild yams (*Dioscorea villosa Linn*) [13], has various properties, such as anti-inflammatory, anti-tumor and antihypercholesterolemia [14,15]. Meanwhile, Dios, a compound found in food, is safe and has no side effects [14]. Dios can inhibit the proliferation and induce apoptosis in various type of tumor cells, and the anti-tumor mechanism of Dios warrants further study [16,17]. Sakshi [18] found that Dios could attenuate tumor growth and metastasis by negatively regulating both NF-κB/STAT3 signaling cascades. Li [19] proved that Dios could exert anti-tumor effects through inactivation of cAMP/PKA/CREB signaling pathway in colorectal cancer. Some studies also showed that Dios could effectively alleviate the side effects of chemotherapeutic drugs, such as decreasing the cardiotoxicity of DOX [20]. We also found the synergistic effect of DOX and Dios on HepG2 cells, which proved that a combination of DOX and Dios would result in an improved treatment of liver cancer and warrants further study.

However, there are some problems in the combination of different drugs, such as the difference of metabolic distribution, and the inability to reach simultaneously the tumor siteto achieve the synergy. Nanoparticle technique is a good choice for current combination therapy and can improve permeability, retention, and pharmacokinetic profiles to avoid some side effects [21]. Liposome as a nano-delivery system is commonly used for the simultaneous loading of different drugs to augment the therapeutic index of the drugs [22,23]. In addition, PEGylated liposomes have been supposed to prolong drug circulation and enhance extravasation in the solid tumor [24]. In liposomes, cholesterol is an essential membrane material, which can regulate the fluidity and stability of lipid bilayer membranes, and maintain high drug entrapment efficiency [25,26]. However, excessive intake of cholesterol at the tumor site will inhibit the anticancer activity of T cells to affect the immune function of the tumor microenvironment [27]. Interestingly, due to the amphiphilic structure of steroidal saponin compounds, many saponins were used as adjuvants to adjust the cell membrane fluidity or permeability by forming complexes with membrane cholesterol [28]. Dios also has a similar structure to cholesterol. It has been proved that Dios has similar membrane regulation because of its similar structure to that of cholesterol, and it has been suggested that Dios (below 30 mol%) has similar effects to cholesterol on basic bio-membrane properties [29]. Therefore, we tried to replace cholesterol with Dios to develop new diosgenin-based liposomes, which further encapsulated DOX to achieve the synergistic treatment of liver cancer.

**Figure 1:**
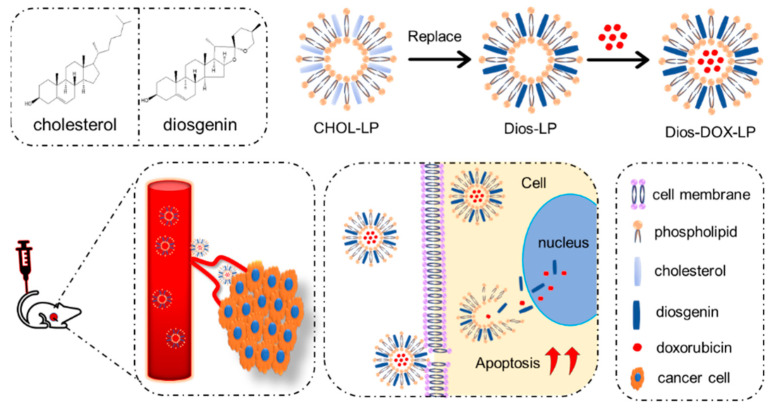
The design of a diosgenin-based liposome delivery system combined with doxorubicin for liver cancer therapy.

In this study, based on the antitumor activities and physicochemical properties of Dios, we prepared a stable and effective Dios-based liposome for the first time, in which Dios substituted cholesterol to avoid high cholesterol in the tumor environment, and was used as a membrane material to form liposomes. As shown in Figure 1, Dios is not only a membrane regulator but also the chemotherapy adjuvant of DOX. Dios-based liposomes improve the solubility of DOX and Dios in water and can accumulate efficiently at the tumor sites during blood circulation. Meanwhile, Dios can increase the antitumor effect of DOX, and improve the deficiency of DOX, such as weight loss and cardiotoxicity, which is useful for the combination treatment in liver cancer.

## 2. Materials and Methods

### 2.1. Materials

Cholesterol, distearoyl phosphocholine (DSPC) and DSPE-mPEG2000 were purchased from AVT Pharmaceutical Co., Ltd. (Shanghai, China). Diosgenin and doxorubicin hydrochloride were purchased from Yuanye Biotechnology Co., Ltd. (Shanghai, China). ICG was purchased from Sanen Chemical Technology Co., Ltd. (Shanghai, China). HepG2 cell line was obtained from the Chinese Academy of Sciences Cells Bank (Shanghai, China). CCK-8 Assay Kit was purchased from Beyotime Biotechnology Co., Ltd. (Shanghai, China). Annexin V-APC Apoptosis Detection Kit was purchased from KeyGen Biotechnology Co., Ltd. (Nanjing, China). BALB (Bagg Albino)/c nude mice were bought from Shanghai SIPPR-Bk Lab Animal Co., Ltd. (Shanghai, China), with approval file No. PZSHUTCM200724024.

### 2.2. In Vitro Synergistic Effect of DOX and Dios on HepG2 Cell

The cytotoxicity and synergistic inhibitory effect of DOX and Dios (the molar ratios of DOX to Dios were 1:1, 1:2, 1:4, 1:6, 1:8, 1:10, 1:15, 1:20, and 1:40, and at least five concentration gradients were set for each ratio) to HepG2 cells were evaluated by CCK-8 assay. Cells (1 × 10^4^ cells/well) were seeded into 96-well micro-plates in MEM medium for 24 h, and then treated with various concentrations of free drugs for 48 h. Then the cells were incubated by CCK-8 for 1 h at 37 °C. The optical density (OD) values were measured at 450 nm with a microplate spectrophotometer (BioTek Instruments, Inc., Winooski, VT, USA). The cell viability was calculated as follows: Cell viability (%) = (ODexperimental sample-Odblank)/(Odcontrol-Odblank) × 100%. the half-maximal inhibitory concentration (IC_50_) values were calculated by SPSS. The combination index (CI) of two drugs was calculated as: CI = [(D)1/(Dx)1] + [(D)2/(Dx)2]. D1 and D2 are the respective concentrations required to produce a certain effect when two drugs are combined. (Dx)1 and (Dx)2 are the concentrations required to produce the same effect when the two drugs are used alone. When CI < 1, it indicates that the two drugs have a synergistic effect, otherwise, it does not.

### 2.3. Preparation of Liposomes

Liposomes were prepared by thin-film dispersion combined ammonium sulfate gradient method [30]. Conventional cholesterol liposomes (CHOL-LP) were prepared with a formulation of DSPC: cholesterol: DSPE-mPEG2000 at a molar ratio of 56:39:5, which referred to Doxil^®^ and Caelyx^®^ [31,32]. Diosgenin liposomes (Dios-LP) were prepared with DSPC: Dios: DSPE-mPEG2000 in a molar ratio of 56:39:5, which replaces the cholesterol with Dios. First, all lipid materials were dissolved in chloroform in a round-bottomed flask and dried using a rotary evaporation apparatus at 60 °C (water bath) to form a thin film. After the thin film was hydrated in ammonium sulfate solution (250 mmol/mL) at 60 °C for 1 h, the liposomal suspension was sonicated by an SCIENTZ-IID ultrasonic processor (Ningbo Scientz Biotechology Co., Ltd., Ningbo, China) in an ice bath to form the liposome. The concentration gradient of liposome was formed after dialysis. Because ammonium sulfate solution was used as the hydration medium, DOX-loaded liposomes could be prepared by adding the appropriate amount (≤2 mg/mL) of DOX into the CHOL-LP and Dios-LP separately and incubating at 60 °C (water bath) for 30 min, which used the concentration gradient of ammonium sulfate in liposomes as the driving force to drive DOX into the aqueous core of liposomes, obtaining CHOL-DOX-LP and Dios-DOX-LP. Finally, the liposome was stored at 4 °C.

### 2.4. Characterization

The particle size, polydispersity index (PDI) and zeta potential of liposomes were measured by a Malvern Zetasizer ZEN3600 Nano ZS (Malvern Instruments, Malvern, Malvern, UK) at 25 °C. The morphology of liposomes was observed by a transmission electron microscope (TEM-1400 plus, JEOL, Tokyo, Japan), after staining with 2% phosphotungstic acid solution. The encapsulation efficiency (EE) and drug loading (DL) of DOX and Dios in the liposomes were determined by mini-column centrifugation-HPLC, where Sephadex G-50 was used to remove the free drugs of liposomes, and liposomes were broken with methanol and analyzed after centrifugation. To evaluate the stability of two liposomes, the change of particle size, PDI and EE were measured and compared for 30 days.

### 2.5. In Vitro Release of DOX and Dios from Liposomes

The in vitro release [33] of DOX and Dios from different formulations was tested with a dialysis method. The dialysis bag has a molecular weight interception of 6000–8000 Da (Yuanye Biotechnology, Shanghai, China). 1 mL of free DOX solution, CHOL-DOX-LP and Dios-DOX-LP, respectively, were loaded into the dialysis bag and placed in 250 mL release medium (normal saline) agitated with a magnetic stirrer at 37 °C for 48 h. The concentration of DOX and solution volume in the bag was measured at the appointed time (0, 0.5, 1, 2, 4, 8, 12, 24, 48 h) to calculate the cumulative release rate. The in vitro release of Dios from different formulations was tested in a 1% Sodium dodecyl sulfate solution as release medium.

### 2.6. In Vitro Cell Cytotoxicity Assay

The cytotoxicity of DOX, CHOL-LP, Dios-LP, CHOL-DOX-LP and Dios-DOX-LP to HepG2 cells was also evaluated using CCK-8 assay. The cells were seeded as in the previous section and then treated with various concentrations of free drugs and liposomes for 48 h. The cells were then incubated with CCK-8 for 1 h at 37 °C to measure the OD values. The cell viability and IC_50_ were calculated by SPSS.

### 2.7. In Vitro Cell Apoptosis Assay

The effects of Dios-LP, DOX, CHOL-DOX-LP and Dios-DOX-LP on inducing apoptosis were detected by flow cytometer (Beckman, Cytoflex S, Brea, CA, USA) [34]. The cells would interfere with Annexin V-FITC fluorescence after DOX administration, when flow cytometry detected. So, Annexin V-APC was chosen to investigate the apoptotic effect of DOX. HepG2 cells were cultured for 24 h and treated with Dios-LP, DOX, CHOL-DOX-LP and Dios-DOX-LP (equal concentration of each DOX and Dios) for 48 h. The cells were then washed by PBS twice and stained with Annexin V-fluorescein APC and 7-AAD successively for 15 min. The percentage of apoptotic cells was quantified by a flow cytometer.

### 2.8. In Vivo Near-Infrared Imaging and Biodistribution

An in vivo near-infrared imaging system was used to evaluate the biodistribution and the targetability of CHOL-DOX-LP and Dios-DOX-LP [35]. CHOL-ICG-LP and Dios-ICG-LP were prepared using the previous method in “2.3”. Nude mice were injected subcutaneously with HepG2 cell suspension (1 × 10^7^ cells/100 μL) into the armpits (right sides) to obtain the tumor bearing model. The mice were randomly divided into 4 groups: normal saline, free ICG, CHOL-ICG-LP and Dios-ICG-LP. Administration was through the tail vein. Using the saline group as a control, the distribution of ICG in vivo and the accumulation in the tumor of free ICG, CHOL-ICG-LP and Dios-ICG-LP, respectively, were monitored at 1 h, 4 h, 8 h and 24 h by an IVIS Lumina XR Imaging System (PerkinElmer Inc., Waltham, MA, USA) (excitation of 745 nm). Finally, the mice were sacrificed after 24 h. The tumor, heart, liver, kidney, lung, and spleen were excised and imaged at the same laser wavelength.

### 2.9. In Vivo Antitumor Activity

The tumor-bearing mouse model was prepared by subcutaneous injection of HepG2 cell suspension (1 × 10^7^ cells/100 μL) into the armpits (right-hand side) of the mice. The mice were randomly divided to four groups when the tumor volume reached about 100 mm^3^. The four groups included a normal saline group, a Dios-LP group (Dios dose = 15 mg/kg), a CHOL-DOX-LP group (DOX dose = 2.5 mg/kg) and a Dios-DOX-LP group (DOX dose = 2.5 mg/kg, Dios dose = 15 mg/kg). The mice were administered every other day and the weight and tumor volumes were measured at the same time. Tumor volume was determined by the vernier caliper and calculated as follow: V (mm^3^) = length × (width)^2^/2. And tumor inhibitory rate was calculated as: tumor inhibitory rate (%) = (W_C_ − W_D_)/W_C_ × 100%, where W_C_ and W_D_ represent the mean weights of tumors in the control group and each administration group, respectively. The mice were sacrificed after 12 administrations. The tumor, heart, liver, kidney, lung, and spleen were collected and weighed after sacrificing. The tissues were all fixed with 4% paraformaldehyde solution, followed by paraffin embedding. Finally, the tissues in different groups were sliced and stained with ki67 and hematoxylin-eosin (HE) to evaluate the effect of drugs on tumor proliferation and safety [36].

### 2.10. Statistical Analysis

All experimental data are presented as mean ± standard deviation (SD). One-way analysis of variance was performed by SPSS to determine the statistical significance of differences among groups. Statistical significance was set at *p* < 0.05.

## 3. Results

### 3.1. Preparation and Characterization of Liposomes

According to the prescription and preparation method of Doxil^®^ and Caelyx^®^, we prepared cholesterol containing liposomes (CHOL-LP) and Dios liposomes (Dios-LP) with a molar ratio at 56:39:5 of DSPC: CHOL/Dios: DSPE-mPEG2000, and encapsulated DOX with two kinds of liposomes to obtain CHOL-DOX-LP (as commercial DOX liposome) and Dios-DOX-LP. Meanwhile, we tried to encapsulate DOX with different concentrations (0.5, 1.0, 2.0 mg/mL) by using Dios-LP, and the characterization of liposomes is shown in Table 1. It can be seen that the entrapment efficiency was high enough and slightly decreased with the increase of DOX concentration, while the difference of particle size and PDI was not significant.

**Figure 2:**
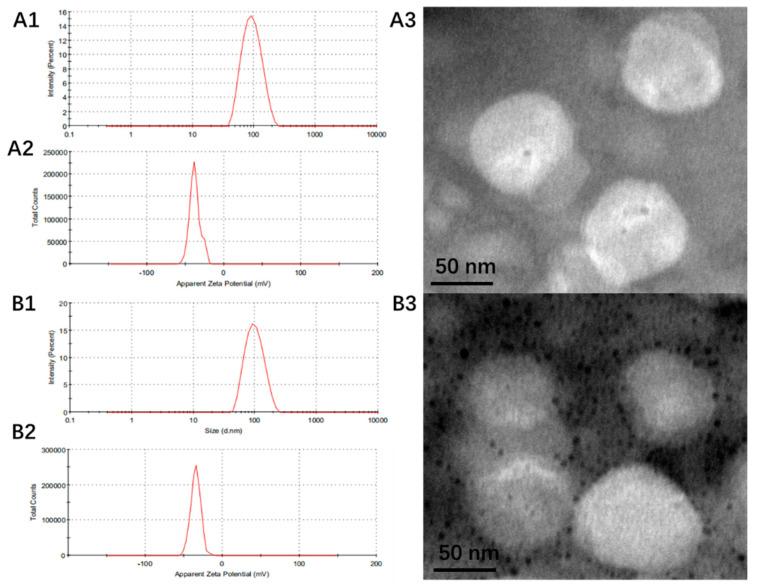
The particle size (**A1**), Zeta (**A2**) and the morphology (**A3**) of CHOL-DOX-LP; The particle size (**B1**), Zeta (**B2**) and the morphology (**B3**) of Dios-DOX-LP.

According to the synergistic ratio in cell cytotoxicity and the results of entrapment efficiency, we selected 0.5 mg/mL DOX (drug/lipid ratio was 1:30 *w*/*w*) as the final liposome preparation. The characterizations are shown in Table 2 and Figure 2. The particle size of Dios-DOX-LP was 99.4 ± 6.2 nm, the PDI was 0.12 ± 0.03, and the Zeta potential was −33.3 ± 2.5 mV. The characterizations of CHOL-DOX-LP and Dios-DOX-LP were similar. The EE of DOX in CHOL-DOX-LP and Dios-DOX-LP was 93.81 ± 0.53% and 98.77 ± 2.04%, respectively, which showed the high encapsulation efficiency of DOX in our liposomes. Meanwhile, CHOL-DOX-LP and Dios-DOX-LP had the similar size and spherical morphology when observed under the electron microscope. Taking the characterization of liposomes as the standard, we verified that loading with Dios rather than cholesterol didn’t significantly impact the characterization of liposomes. Dios could still maintain the uniform particle size and high entrapment efficiency of liposomes, which preliminarily confirmed the cholesterol-like ability of Dios in liposomes.

### 3.2. In Vitro Leakage and Release of DOX from Liposomes

**Figure 3:**
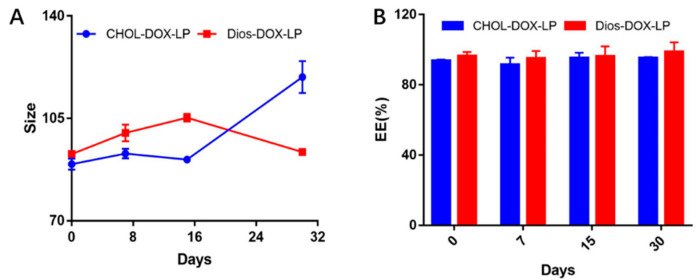
(**A**)The comparison of particle size between CHOL-DOX-LP and Dios-DOX-LP in 30 days; (**B**) The comparison of EE between CHOL-DOX-LP and Dios-DOX-LP in 30 days.

The stability of two DOX loaded liposomes was investigated based on the particle size and EE within a month, as shown in Figure 3. Within 30 days, the particle size of both liposomes varied from 90 nm to 110 nm, and the PDI were all less than 0.30. As for EE of DOX, the EE of CHOL-DOX-LP was approximately 93% to 95%, and that of Dios-DOX-LP was around 98%. There was no obvious drug leakage in either liposome in 30 days, which showed that the liposomes obtained by replacing cholesterol with diosgenin were still stable.

**Figure 4:**
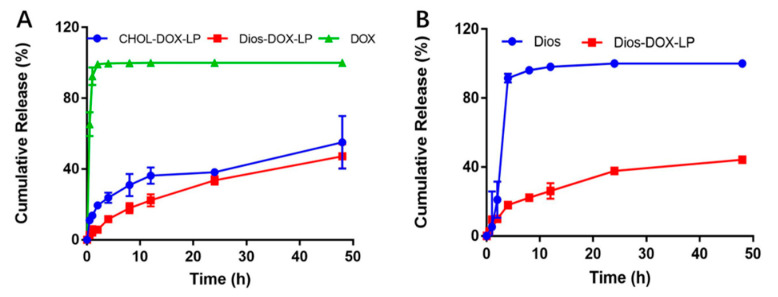
(**A**) In vitro cumulative release of DOX in free DOX, CHOL-DOX-LP and Dios-DOX-LP; (**B**) In vitro cumulative release of Dios in free Dios and Dios-DOX-LP.

We compared and characterized the in vitro cumulative releases of DOX and Dios which are shown in Figure 4. After being dialyzed for 48 h, CHOL-DOX-LP and Dios-DOX-LP showed the cumulative release of DOX (55.05% and 47.22%, respectively), which was clearly lower than that of free-DOX. This illustrated that the liposomal bilayer could effectively slow down the DOX release to produce a favorable sustained-release capability. Meanwhile, the release curves of CHOL-DOX-LP and Dios-DOX-LP were similar. The cumulative releases of Dios also showed the sustained-release effect of Dios-DOX-LP. Dios-DOX-LP showed the Dios cumulative release of 44.26% after 48 h, which was closed to that of DOX, and achieved synchronous release. The result suggested that Dios interacted with the liposomal membrane to ensure that DOX was not easy to leak. In view of the particle size and EE, and the stable and sustained-release, liposomes can be obtained by replacing cholesterol with Dios in equal proportion, which provides data support for the idea of a novel Dios-based liposome delivery system.

### 3.3. In Vitro Cytotoxicity

**Figure 5:**
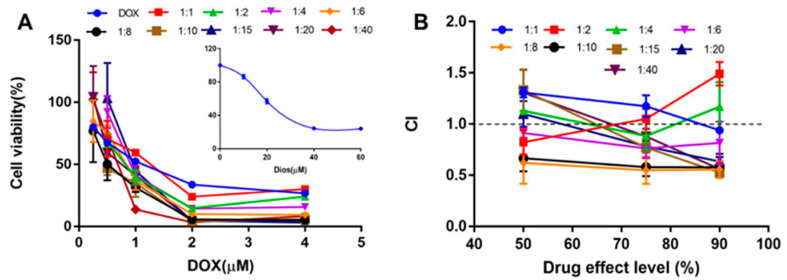
The synergistic effect of DOX and Dios on HepG2 cells. (**A**) The cell viability of different proportions of DOX + Dios on HepG2 cells; (**B**) CI values of DOX + Dios in different proportions.

The antitumor activity of DOX, Dios and DOX + Dios was first evaluated by CCK-8 assay in vitro. The IC_50_ of DOX and Dios were 1.03 ± 0.12 μM and 28.51 ± 2.44 μM, respectively, and exhibited dose-dependent cytotoxic activity. The cell survival curves of DOX, Dios and drug combination were shown in Figure 5A, and the CI values were shown in Figure 5B. When the molar ratios (DOX: Dios) were 1:6, 1:8 and 1:10, the CI was lower than 1 at the inhibition rates 50%, 75% and 90%. While the CI values of other drug combinations were higher than or equal to 1. Therefore, the DOX: Dios molar ratio has a synergistic effect in the range of 1:6 to 1:10, that is, the DOX: Dios mass ratio ranges from 1:4.29 to 1:7.15 (*w*/*w*). DOX and Dios showed synergy at some molar ratio, which provides a basis for subsequent combination of two drugs.

**Figure 6:**
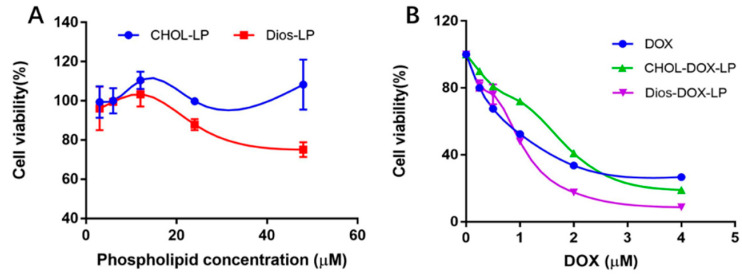
(**A**) The effect of CHOL-LP and Dios-LP on HepG2 cells; (**B**) The effect of different concentrations of DOX, CHOL-DOX-LP, and Dios-DOX-LP on HepG2 cells.

The cytotoxicity of DOX, Dios, CHOL-LP, Dios-LP, CHOL-DOX-LP and Dios-DOX-LP to HepG2 cells is shown in Table 3 and Figure 6. CHOL-LP as blank liposome did not inhibit the growth of HepG2 cells, and the cell survival rate was always around 100% as the phospholipid concentration changed, while the IC_50_ of Dios-LP was 33.93 ± 2.47 μM, as shown in Figure 6A. This meant that replacing the prescribed cholesterol with Dios gave the liposomes themselves an antitumor effect. DOX, CHOL-DOX-LP and Dios-DOX-LP led to a significant reduction in cell viability. The IC_50_ of DOX in DOX, CHOL-DOX-LP and Dios-DOX-LP were 1.03 ± 0.12 μM, 1.42 ± 0.02 μM and 0.87 ± 0.08 μM, separately. The cytotoxicity of Dios-DOX-LP was higher than that of free drug and CHOL-DOX-LP, which was attributed to the synergistic effect of DOX and Dios in Dios-DOX-LP. This combination preliminarily proved that the anticancer of DOX was amplified in vitro by Dios-DOX-LP.

### 3.4. In Vitro Cell Apoptosis Assay

**Figure 7:**
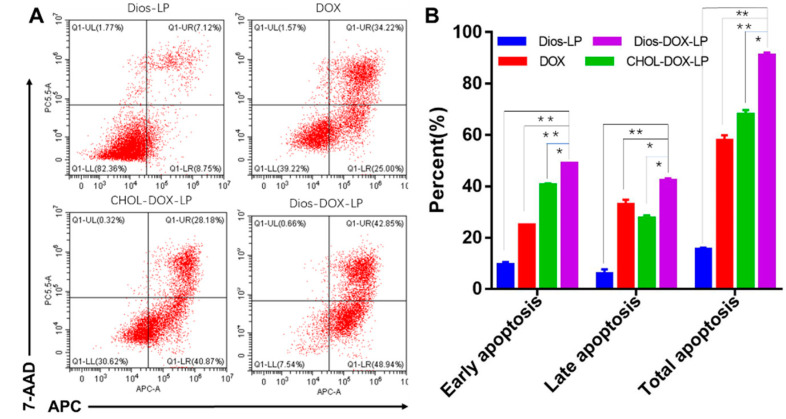
(**A**) Apoptosis induced by Dios-LP, DOX, CHOL-DOX-LP, and Dios-DOX-LP on HepG2 cells; (**B**) Results of apoptotic percent on HepG2 cell induced by Dios-LP, DOX, CHOL-DOX-LP, and Dios-DOX-LP (*: there was significant difference, *p* < 0.05; **: there was significant difference, *p* < 0.01).

The results of the apoptosis experiment are shown in Figure 7. The dose of Dios in Dios-LP was equal to that in Dios DOX LP, and Dios could promote apoptosis, but was not strong enough. According to the percentage of total apoptotic cells, the apoptosis-inducing effects of DOX and CHOL-DOX-LP were 57.72 ± 2.13% and 67.97 ± 1.68%, respectively, while the effects of Dios-DOX-LP were significantly improved to 91.01 ± 1.11%. Meanwhile, the percentage of early apoptosis and late apoptosis were both increased with the addition of Dios. Apoptosis is the ultimate embodiment of antitumor effects [37]. After encapsulating DOX and Dios for simultaneous administration, Dios enhanced early and late apoptosis of DOX. The results were consistent with the trend of cytotoxicity, which further explained that Dios could synergize with DOX by Dios-DOX-LP in killing tumor cells.

### 3.5. In Vivo Near-Infrared Imaging and Biodistribution

**Figure 8:**
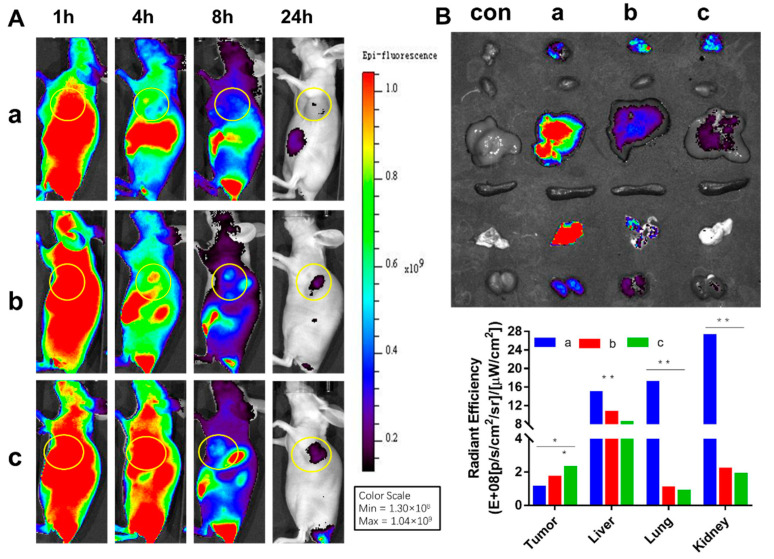
(**A**) The in vivo biodistribution imaging of HepG2 tumor-bearing mice; (**B**) The biodistribution imaging of tumor and representative organs; **a**: ICG; **b**: CHOL-ICG-LP; **c**: Dios-ICG-LP. (*: there was significant difference, *p* < 0.05; **: there was significant difference, *p* < 0.01).

The results of in vivo near-infrared imaging and biodistribution are shown in Figure 8A. The three groups of ICG, CHOL-ICG-LP and Dios-ICG-LP showed different distributions in vivo. The fluorescence of free ICG in mice decreased over time after administration and the ICG did not gather or remain at the tumor site. In both the CHOL-ICG-LP group and Dios-ICG-LP group, ICG distributed throughout the body and accumulated subsequently at the tumor site, which showed the targeting ability of liposomes. Meanwhile, the fluorescence decreased slower in the Dios-ICG-LP group than in the CHOL-ICG-LP group. The ICG fluorescence intensity at the tumor site and other representative organs after 24 h are shown in Figure 8B. In the free ICG group, the fluorescence in the tumor could be barely detected, and there were obvious fluorescence distributions in the liver, lungs and kidneys. However, the fluorescence at the tumor site was stronger in the two liposome groups, and there was no obvious fluorescence in other organs. The targeting ability of Dios-ICG-LP was slightly better than that of CHOL-ICG-LP. The in vivo imaging results demonstrated that the Dios-based liposome also had an EPR effect, which was conducive to escaping from the reticuloendothelial system and passively targeting tumors, resulting in high tumor accumulation [38]. Definitely, the Dios-based liposome potentiated the efficacy of the tumor targeting of drugs, and reduced systemic toxicity.

### 3.6. In Vivo Antitumor Activity

**Figure 9:**
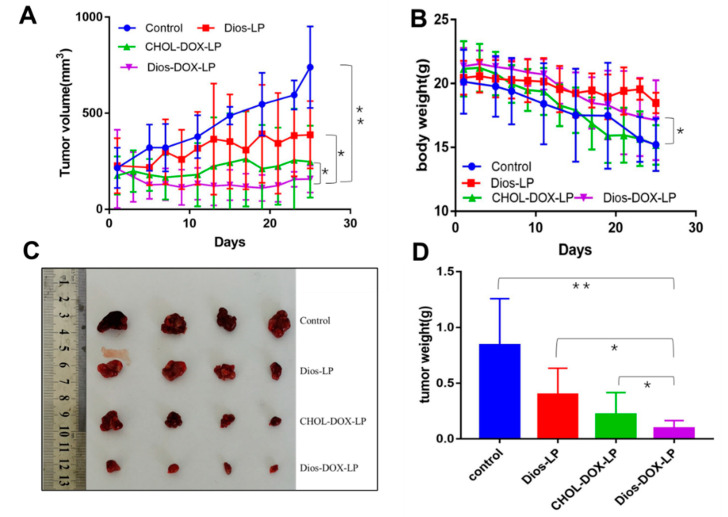
(**A**) Tumor volume changes in each group; (**B**) Body weight change in each group; (**C**) Tumor size in each group; (**D**) Tumor weight of each group. *: there was significant difference, *p* < 0.05; **: there was significant difference, *p* < 0.01 (n = 4).

**Figure 10:**
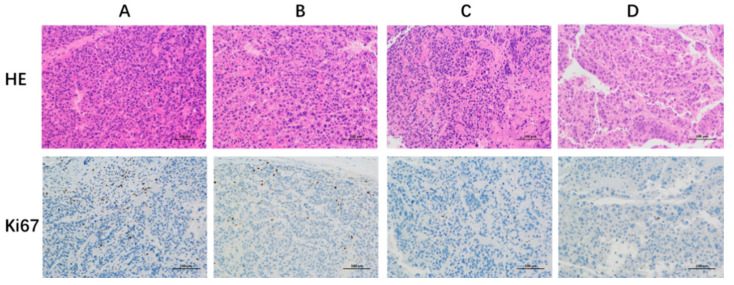
The HE and Ki67 staining results of the tumor tissues at 200× magnification with the optical microscope. (**A**) control group; (**B**) Dios-LP group; (**C**) CHOL-DOX-LP group; (**D**) Dios-DOX-LP group.

The antitumor effect of liposomes in vivo was investigated. The changes of tumor volume were shown in Figure 9A,C,D. All three groups of liposomes (Dios-LP, CHOL-DOX-LP, Dios-DOX-LP) showed certain antitumor effects when compared with the control group. The tumor inhibition rate of Dios-LP, CHOL-DOX-LP and Dios-DOX-LP groups were approximately 47.6%, 66.4% and 78.7%, respectively, suggesting that the antitumor effect of Dios-DOX-LP was significantly improved. Moreover, larger shaped nuclei were observed in the H&E and ki67 stained sections of the tumor tissue in the control group, which indicates cell proliferation, while the tumors treated with Dios-DOX-LP were observed with scattered nuclei and cytoplasmic vacuolation, which indicated tumor tissue necrosis (Figure 10). The synergistic effect of Dios and DOX in Dios-DOX-LP was also confirmed in vivo.

**Figure 11:**
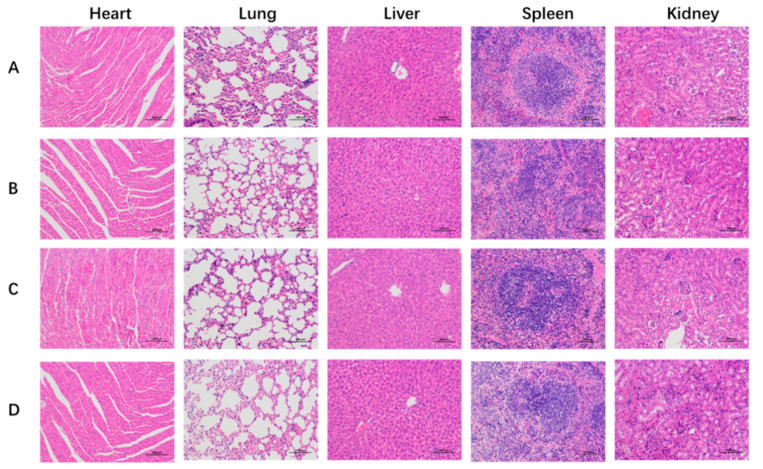
The histological characteristics of representative organs at 200× magnification with the optical microscope. (**A**) control group; (**B**) Dios-LP group; (**C**) CHOL-DOX-LP group; (**D**) Dios-DOX-LP group.

Toxicities were also estimated by the change in body weight and the H&E staining of major organs in mice. As shown in Figure 9, the body weights were monitored for 25 d, and the treatment of Dios-DOX-LP did not obviously affect the body weights when compared with the control group, indicating that diosgenin-based liposome can reduce the toxicity and increase the efficiency of DOX, reflecting the replacement advantages of Dios. The H&E staining images of main organs (heart, liver, lungs, spleen, and kidneys) from the different groups in Figure 11 shows that Dios-LP, CHOL-DOX-LP and Dios-DOX-LP had no obvious tissue toxicity in vivo. In general, Dios-DOX-LP had a good, safe antitumor effect.

## 4. Discussion

The study developed a novel Dios-based liposome in combination with DOX for liver cancer therapy for the first time. Dios-DOX-LP had uniform particle size, good stability, high entrapment efficiency and a slow-release effect, and was similar to CHOL-DOX-LP. We also demonstrated that Dios-DOX-LP synergistically inhibited cell proliferation and induced apoptosis to achieve better antitumor effects than CHOL-DOX-LP in vivo and in vitro, in which Dios also had synergistic effects and no side effects to ensure the safety and effectiveness of liposome. The major advantage of the Dios-based liposomes was to achieve toxicity reduction and efficiency enhancement of chemotherapeutic drugs. There are many studies on the combination of natural products and chemotherapy drugs [39]. However, unlike diosgenin, it is difficult for most nature products to be used as medicine and adjuvant at same time. Meanwhile, the production technology of diosgenin is mature and low cost, which makes it more applicable in pharmaceutical industry [13]. In the future, the Dios-based liposomes can load other drugs to achieve a better antitumor effect, which can in turn provide a safer and more efficient chemotherapeutic drug liposome delivery for the clinical treatment of different tumors.

## Figures and Tables

**Table 1:** Effects of DOX with different concentrations on particle size, potential and entrapment efficiency of liposomes of Dios-DOX-liposomes.

DOX/Lipid Ratio (*w*/*w*)	Size (nm)	PDI	EE (%)
1:30	99.4 ± 6.2	0.12 ± 0.03	98.77 ± 2.04
2:30	101.5 ± 4.6	0.17 ± 0.04	100.17 ± 6.86
4:30	98.6 ± 4.6	0.15 ± 0.03	86.37 ± 2.35

**Table 2:** Comparison of particle size, PDI, Zeta, EE and DL between CHOL-LP, CHOL-DOX-LP, Dios-LP and Dios-DOX-LP.

Liposome	Size (nm)	PDI	Zeta (mV)	EE (%)		DL (%)	
				DOX	Dios	DOX	Dios
CHOL-LP	88.8 ± 5.5	0.24 ± 0.03	−33.8 ± 5.2	/	/	/	/
CHOL-DOX-LP	90.3 ± 4.8	0.16 ± 0.02	−38.4 ± 0.8	93.81 ± 0.53	/	2.54 ± 0.01	/
Dios-LP	98.2 ± 3.4	0.10 ± 0.02	−33.8 ± 0.8	/	91.47 ± 4.27	/	14.83 ± 0.69
Dios-DOX-LP	99.4 ± 6.2	0.12 ± 0.03	−33.3 ± 2.5	98.77 ± 2.04	87.75 ± 2.93	2.67 ± 0.55	14.23 ± 0.47

**Table 3:** IC_50_ of DOX, Dios-LP, CHOL-DOX-LP and Dios-DOX-LP (µM).

IC_50_	DOX	Dios-LP	CHOL-DOX-LP	Dios-DOX-LP
DOX	1.03 ± 0.12	/	1.42 ± 0.02	0.87 ± 0.08 *
Dios	/	33.93 ± 2.47	/	5.20 ± 0.48

*: compared with DOX, there was significant difference, *p* < 0.05.

## Data Availability

The data presented in this study are available on request from the corresponding author.
